# AUXIN-BINDING-PROTEIN1 (ABP1) in phytochrome-B-controlled responses

**DOI:** 10.1093/jxb/ert294

**Published:** 2013-09-19

**Authors:** Yunus Effendi, Alan M. Jones, Günther F. E. Scherer

**Affiliations:** ^1^Leibniz Universität Hannover, Institut für Zierpflanzenbau und Gehölzforschung, Abt. Molekulare Ertragsphysiologie, Herrenhäuser Str. 2, D-30419 Hannover, Germany; ^2^Departments of Biology and Pharmacology, University of North Carolina, Chapel Hill, NC 27516, USA

**Keywords:** AUXIN-BINDING PROTEIN1 (ABP1), early auxin-regulated genes, elongation, gravitropism, phototropism, phytochrome, shade avoidance.

## Abstract

The auxin receptor ABP1 directly regulates plasma membrane activities including the number of PIN-formed (PIN) proteins and auxin efflux transport. Red light (R) mediated by phytochromes regulates the steady-state level of ABP1 and auxin-inducible growth capacity in etiolated tissues but, until now, there has been no genetic proof that ABP1 and phytochrome regulation of elongation share a common mechanism for organ elongation. In far red (FR)-enriched light, hypocotyl lengths were larger in the *abp1-5* and *abp1/ABP1* mutants, but not in *tir1-1*, a null mutant of the TRANSPORT-INHIBITOR-RESPONSE1 auxin receptor. The polar auxin transport inhibitor naphthylphthalamic acid (NPA) decreased elongation in the low R:FR light-enriched white light (WL) condition more strongly than in the high red:FR light-enriched condition WL suggesting that auxin transport is an important condition for FR-induced elongation. The addition of NPA to hypocotyls grown in R- and FR-enriched light inhibited hypocotyl gravitropism to a greater extent in both *abp1* mutants and in *phyB-9* and *phyA-211* than the wild-type hypocotyl, arguing for decreased phytochrome action in conjunction with auxin transport in *abp1* mutants. Transcription of FR-enriched light-induced genes, including several genes regulated by auxin and shade, was reduced 3-5-fold in *abp1*-5 compared with Col and was very low in *abp1/ABP1*. In the *phyB-9* mutant the expression of these reporter genes was 5–15-fold lower than in Col. In *tir1-1* and the *phyA-211* mutants shade-induced gene expression was greatly attenuated. Thus, ABP1 directly or indirectly participates in auxin and light signalling.

## Introduction

Auxin initiates responses by at least two different receptors, AUXIN BINDING PROTEIN1 (ABP1) and TRANSPORT INHIBITOR RESPONSE1 (TIR1) (Scherer, 2011). TIR1 mediates auxin effects on gene expression (Mockaitis and Estelle, 2008), while ABP1 mediates auxin effects at the plasma membrane (Napier *et al.*, 2002; Robert *et al.*, 2010; Xu *et al.*, 2010). ABP1 is essential for development and many rapid cellular changes (Jones *et al.*, 1998; Chen *et al.*, 2001*a*, *b*). ABP1-mediated rapid responses such as membrane hyperpolarization, channel regulation, proton extrusion, phospholipase A activation (Scherer and Andrè, 1989; Labusch *et al.*, 2013), phospholipase D activation, transient increase in cytosolic calcium and elongation are too rapid to be reconciled with TIR1 as the only auxin receptor, assuming that the sole function of TIR1 is mediating changes in gene transcription through its degradation of transcriptional regulators (Badescu and Napier, 2006; Scherer, 2011).

ABP1 is a small glycoprotein localized in the ER lumen with 1–3% secreted to the extracytosolic side of the plasma membrane where it binds auxin (Tian *et al.*, 1995; Napier *et al.*, 2002). The *ABP1* expression pattern is strongly overlapping with that of the artificial auxin-activated *DR5* promoter coupled to the *uidA* gene (Klode *et al.*, 2011) suggesting a causal relationship between ABP1 action and auxin concentrations, consistent with the observation that auxin regulates *ABP1* transcription (Hou *et al.*, 2006; Effendi *et al.*, 2011). In order to transmit signalling to cytosolic proteins, a transmembrane protein, ‘docking protein’ or binding protein for ABP1, was postulated (Klämbt, 1990). A critical feature of hormone receptors is that the activated pool size limits the amplitude and/or rate of signal transduction at physiological concentrations of the cognate hormone (Kenakin, 2004). Consistent with the ABP1 number being rate-limiting for auxin responses, null *abp1* mutants are embryo lethal (Chen *et al.*, 2001*b*) and the heterozygous *abp1/ABP1* mutant displays auxin-signalling defects (Effendi *et al.*, 2011). It was speculated that proper stoichiometry of ABP1 and the hypothetical binding protein is rate-limiting for signal output and any disturbance of this stoichiometry causes a mutant auxin phenotype. This gene dosage effect or haploinsufficiency (Veitia *et al.*, 2008) is common for receptors in humans (Fisher and Scambler, 1994). A dosage effect for ABP1 function was also demonstrated using conditional deletion by expressing a recombinant antibody fragment directed against ABP1, a line designated *abp1-SS12* (Braun *et al.*, 2008). Additional observations that active ABP1 is rate-limiting are: (i) the level of ABP1 and auxin-induced growth capacity is correlated in tobacco leaves (Chen *et al.*, 2001*b*), (ii) genetic ablation of ABP1 blocks embryogenesis at an early phase when auxin induces the elongation of the top tier of cells (Chen *et al.*, 2001*b*), and (iii) reduction of ABP1 reduces auxin-induced expansion without an effect on auxin-induced cell division (Jones *et al.*, 1998).

Most, if not all, phenotypes associated with *ABP1* mutations are linked to a malfunction of polar auxin transport conducted or regulated by PIN proteins (Robert *et al.*, 2010; Xu *et al.*, 2010; Effendi *et al.*, 2011; Effendi and Scherer, 2011). PIN1 proteins are located on the plasma membranes along the tips of epidermal cell lobes and are linked to the expansion of lobes in an auxin signalling pathway that uses ABP1 as a receptor and small G proteins as intermediates (Xu *et al.*, 2010). At these positions, the level of auxin is critical for the proper development of pavement cells (Xu *et al.*, 2010). Robert *et al.* (2010) showed that ABP1 is the receptor for the auxin-inhibition of endocytosis of PIN proteins. As a consequence, the efflux transport by these PIN proteins is enhanced (Paciorek *et al.*, 2005). Another example of a possible link between ABP1 and polar auxin transport is the correlation of ABP1, auxin concentration, and H^+^-ATPase localization in embryo development (Chen *et al.*, 2010). It was shown, in particular, that the heterozygous T-DNA insertion mutant *abp1/ABP1* has defects in (i) root and hypocotyl gravitropism, (ii) basipetal auxin transport in the root, (iii) apical dominance, and (iv) regulation of early auxin-activated genes (Effendi *et al.*, 2011). In our model, these functions were linked to the regulation of auxin transport which, in turn, regulates the auxin concentrations perceived by the extracytosolic ABP1 receptor and the nuclear receptor TIR1 (Effendi *et al.*, 2011; Effendi and Scherer, 2011; Scherer *et al.*, 2012).

Red (R) and blue (B) light decreases auxin transport, steady-state ABP1 level, and auxin-binding capacity (Shinkle and Jones, 1988; Jones *et al.*, 1991; Shinkle *et al.*, 1992, 1998; Barker-Bridges *et al.*, 1998; Liu *et al.*, 2011). R decreased the steady-state level of ABP1 and auxin transport over a time-course consistent with the kinetics of R-induced decrease in hypocotyl elongation. Other light-regulated physiological responses involve auxin transport and require ABP1. Increased hypocotyl elongation in FR-enriched light, and expression of rapidly R- or FR-induced genes were all different in *abp1-5* and *abp1/ABP1* compared with wild types (wt). Further, impeding elongation and gravitropism in hypocotyls by the auxin transport inhibitor naphthylphenylphthalamic acid (NPA) revealed the impact of auxin transport on these phytochrome-controlled responses as proposed (Robson and Smith, 1996; Jensen *et al.*, 1998; Keuskamp *et al.*, 2010; Kozuka *et al.*, 2010). Thus, ABP1 plays a direct or indirect role in the shade avoidance response in *Arabidopsi*s and it is speculated that ABP1 regulates auxin transport as part of the mechanism.

## Materials and methods

### Plant material and growth conditions

Heterozygous kanamycin-resistant *abp1/ABP1* mutant seeds (Chen *et al.*, 2001*b*) are in a Ws background and the genotypes verified as before (Chen *et al.*, 2001*b*; Effendi *et al.*, 2011). *abp1-5* contains a mutation of a conserved histidine to a tyrosine (H94Y) (Robert *et al.*, 2010) in the auxin-binding pocket of ABP1 (Woo *et al.*, 2002). *phyA-211* and *phyB* are in the Col-0 background and were obtained from M Zeidler,and *tir1-1* and *tir1-9* were obtained from M Quint.

For the gravitropism and phototropism experiments, seeds were stratified for 4 d, treated for 4h with WL and grown for 3 d vertically on 0.5× MS agar plates in the dark at 22.5 °C. For testing gravitropism, plants were turned 90° for 24h and then scanned. Lateral blue light at 10 μmol^.^m^–2.^s^–1^ (CLF, Plant Climatics) was applied and scanned after 8h (CanonScan 8800F; resolution 600 dots per inch). For testing shade avoidance, seeds were stratified for 4 d, treated with WL for 4h, and then kept in the dark for 24h. Thereafter, WL (14.5 μmol m^–2.^s^–1^) was applied for 3 d, followed by WL supplemented with R and FR either with a high R:FR ratio (2.11) or a low R:FR ratio (0.098) in an LED box at 22.5 °C (CLF, Plant Climatics) for another 3 d at 22.5 °C or on NPA-containing agar or 1h for subsequent RNA isolation. Hypocotyl lengths or angles were measured using AxioVision LE Ver.4.6 software (Zeiss-Germany). For flowering time experiments, plants were grown in a growth chamber at 22.5 °C in 8/16h (L/D). Each experiment was done at least twice. Where necessary, heterozygous *abp1/ABP1* plants were identified by genotyping as before (Chen *et al.*, 2001*b*; Effendi *et al.*, 2011).

### Nucleic acid analysis

For transcription measurements, seedlings were grown in 0.5× MS agar-medium for 14 d in long (12/12h) days. For the auxin treatment, the medium was removed and replaced by fresh medium containing 10 μM 1-NAA. Seedlings were blotted on filter paper and frozen in liquid nitrogen for further use. For quantitative RT-PCR, 4 μg of total RNA was prepared with the NucleoSpin^®^ RNA Plant kit according to the manufacturer’s instructions (Macherey and Nagel) and transcribed to first strand cDNA with RevertAid^TM^ H Minus First Strand cDNA Synthesis kit (Fermentas). Primers and methods were as described previously (Effendi *et al.*, 2011; Effendi and Scherer, 2011; the primers are listed in Supplementary Table S1 at *JXB* online). For each data point, two to five biological repeats and three technical replicates for each determination were done in the subsequent PCR reaction. Relative expression was calculated according to the ΔΔCt method using the equation: relative expression=2^–[ΔCtsample–ΔCtcontrol]^, with ΔCt=Ct_sample gene_–Ct_reference gene_, where Ct refers to the threshold cycle determined for each gene in the early exponential amplification phase (Livak and Schmittgen, 2001). The control treatment at *t*=0min was set as 1-fold expression level. For statistical analysis the REST 2008 software (Pfaffl *et al.*, 2002) was used.

## Results

**Fig. 1.**
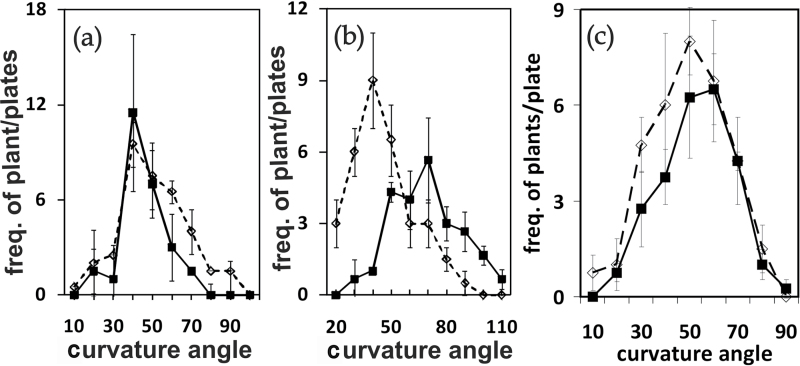
Gravitropic and phototropic responses in 3-d-old dark-grown Col-0 (black squares) and *abp1-5* (diamonds) seedlings. (a) Gravitropic bending angles of hypocotyls after 24h tilting by 90° (mean Col: 44.8°; *n*=57; mean *abp1-5*: 46.7°; *n*=42; *P* <0.54; difference not significant). (b) Gravitropic bending angles of roots after 24h tilting by 90° (mean Col: 65.3°; *n*=71; mean *abp1-5*: 41.1°; *n*=65; *P* <0.001). (c) Phototropic bending angles of hypocotyls after 8h lateral blue light (10 μmol m^–2^ s^–1^) (mean Col: 48.9°; *n*=135; mean *abp1-5*: 45.7°; *n*=102; *P* <0,114; difference not significant). For each panel, 3–4 agar plates containing about 30 seedlings were evaluated. Data points represent means of each angle size group and SE.

The mutant *abp1-5* containing a histidine 94→tyrosine point mutation has near-normal morphology (see Supplementary Fig. S1 and Fig. S2 at *JXB* online; data not shown). As shown in Supplementary Fig. S2 at *JXB* online, both flowering time and the number of rosette leaves at the beginning of flowering were nearly identical in *abp1-5* and in the wild type in short days in contrast to *abp1/AB*P1 (Effendi *et al.*, 2011). Although, the gravitropic response of hypocotyls and the phototropic response to laterally applied blue light of hypocotyls of *abp1-5,* grown in the dark, was statistically indistinguishable from the wild type (Fig. 1a, c), the gravitropic response in roots was less than the wild type (Fig. 1b). *abp1/ABP1* seedlings had an agravitropic and an aphototropic phenotype (Effendi *et al.*, 2011). To a lesser extent as in *abp1*/ABP1 (Effendi *et al.*, 2011), delayed expression of several auxin-inducible genes was found in *abp1-5* (see Supplementary Fig. S3 at *JXB* online) confirming that ABP1 affects auxin function(s).

**Fig. 2.**
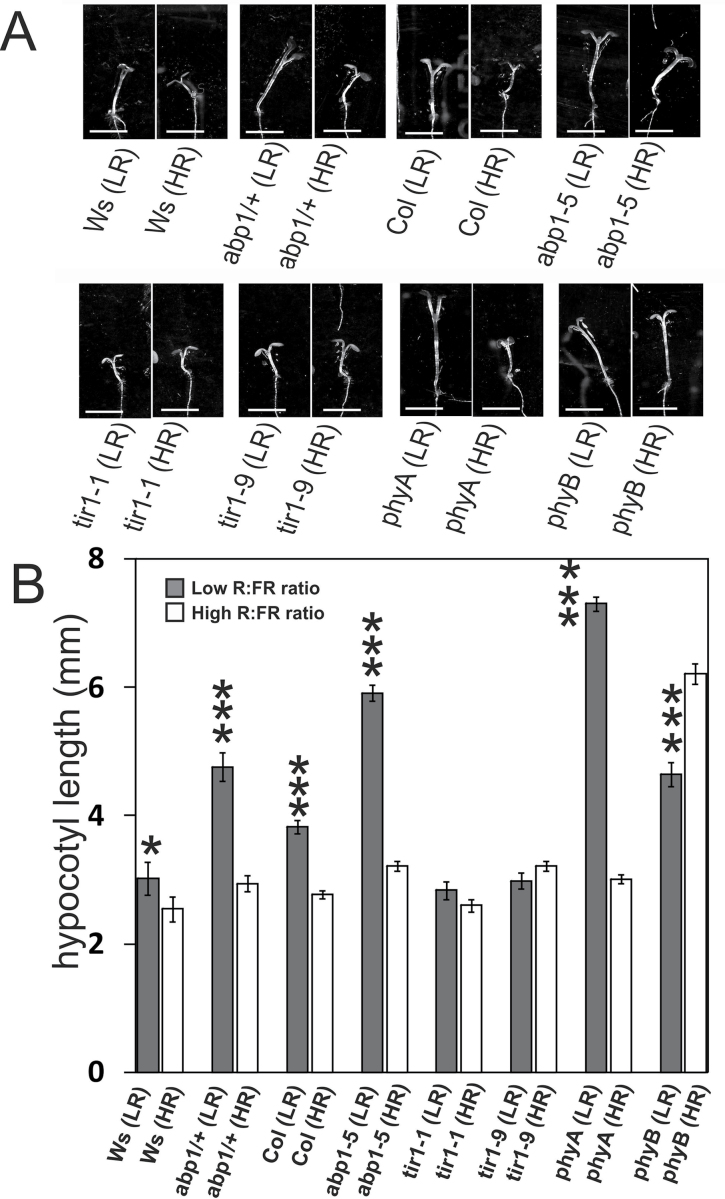
Shade-avoidance responses in *abp1-5* and *abp1/ABP1* compared with Col, *phyA-211*, and *phyB-9*. Shade avoidance was tested by growing seedlings for 3 d in WL and for 3 more days in WL or white plus added low R:FR ratios (LR, simulated shade) or high ratios of R:FR (HR, non-shade). Seedlings from seeds from an *abp1/ABP1* plant were verified by PCR-genotyping as either Ws wild type or *abp1/ABP1* mutant (Effendi *et al.*, 2011). For comparison, *phyA-211* and *phyB-9* mutants were added to the tests. (A) Representative seedlings of every line used grown in low or high ratio of FR:R. Bar=5mm. (B) The hypocotyl lengths of seedlings grown in low (dark bars) or high ratio (white bars) of R:FR. Hypocotyl lengths of seedlings were evaluated. LR and HR treatments were statistically different except for the *tir1* alleles. Significance levels in (B): *P <0.05; **P <0.01; ***P <0.001; (*n*=55–90; SE).

Accelerated hypocotyl elongation is characteristic of the shade avoidance response in plants and depends on auxin transport (Jensen *et al.*, 1998). In both *abp1-5* and in *abp1/ABP1* mutant seedlings, the response to FR-enriched light was tested and compared with the response in *tir1* mutants. Plants were grown first in WL for 3 d and either continued with augmented R light to create a high red:far red (R:FR) ratio (non-shade) or at a low R:FR ratio (shade) for another 3 d (spectra in Supplementary Fig. S4 at *JXB* online). Hypocotyl elongation in both *abp1* mutants were significantly taller in FR-enriched light than in the wild type. The respective wild types showed a much smaller elongation response to low R:FR (Fig. 2). In high R:FR ratio conditions, the *abp1* mutants were like the wild type.

TIR1 regulates gene transcription by auxin-stimulated ubiquitination of AUX/IAA proteins which are negative co-transcription factors (Mockaitis and Estelle, 2008). Therefore, two *tir1* alleles, *tir1-1* and *tir1-9*, were also tested for their elongation response to shade conditions (Fig. 2). In contrast to *abp1* mutants, hypocotyl lengths of *tir1-1* and *tir1-9* in both low and high R:FR conditions were not significantly different and they exhibited no shade response.

**Fig. 3.**
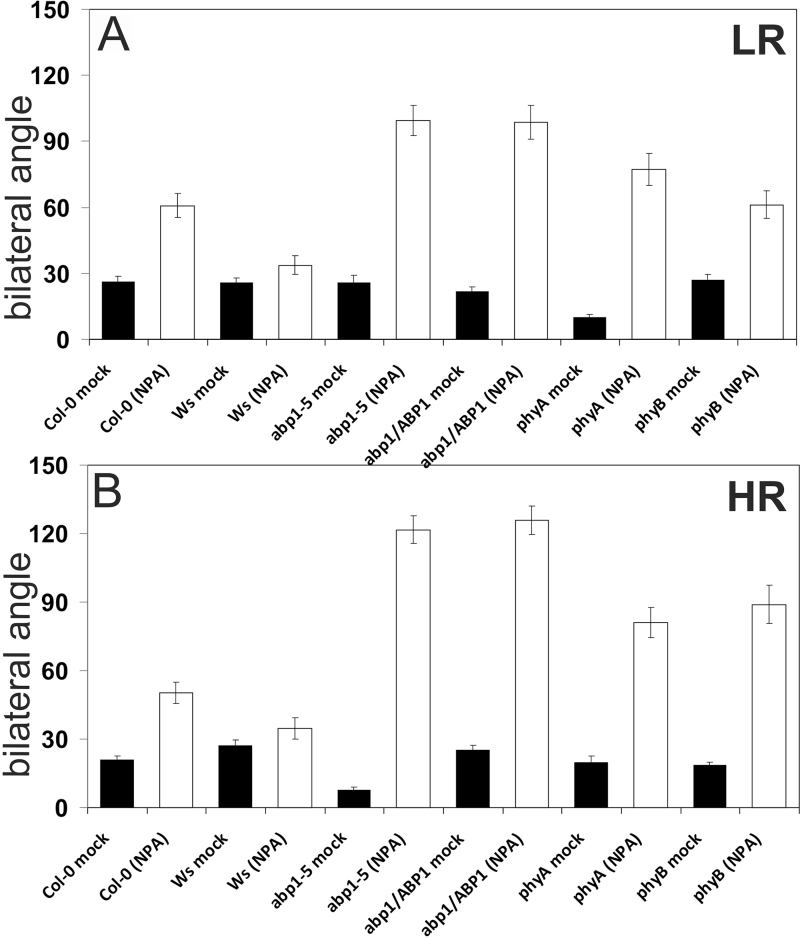
Diagnostic effects of 5 μM NPA on hypocotyl gravitropic orientation in (A) low and (B) high ratio R:FR light in *abp1-5* and *abp1/ABP1* and phytochrome mutants and wild types. Data are from 24 to 54 seedlings per assay (SE). The genotype of *abp1/ABP1* plants was verified by PCR. In LR Col and *phyB* seedlings in the presence of NPA were not statistically significant different but *phyA* seedlings were different from Col (*P* <0.05). In HR, all mutants in the presence of NPA were significantly different from the wild types (*P* <0.01 or lower).

R and FR abrogate hypocotyl gravitropism and the inhibition of hypocotyl gravitropism depends on active P_r_ of either phyA or phyB (Liscum and Hangarter, 1993; Robson and Smith, 1996) and NPA, originally described as a gravitropic inhibitor (Geissler *et al.*, 1985), has become a diagnostic tool for auxin transport. As shown in Fig. 3, *abp1* mutants and phytochrome mutants lose their gravitropic orientation in both low and high R:FR (*P* <0.01) with the exception of *phyB* in low ratio R:FR light (versus Col) and the effect of NPA was similar on *abp1* and *phy* mutants. The effect of NPA on elongation induced in low R:FR light was also tested (Jensen *et al.*, 1998; Steindler *et al.*, 1999; Kozuka *et al.*, 2010) and it was compared with the effect of NPA on elongation in high ratio R:FR light in the *abp1* mutants and *phyA* and *phyB* mutants (see Supplementary Fig. S5 at *JXB* online). Greater NPA inhibition was simply associated with taller hypocotyls, a sensitivity difference in mutants or wild types to NPA concentration was small if any.

**Fig. 4.**
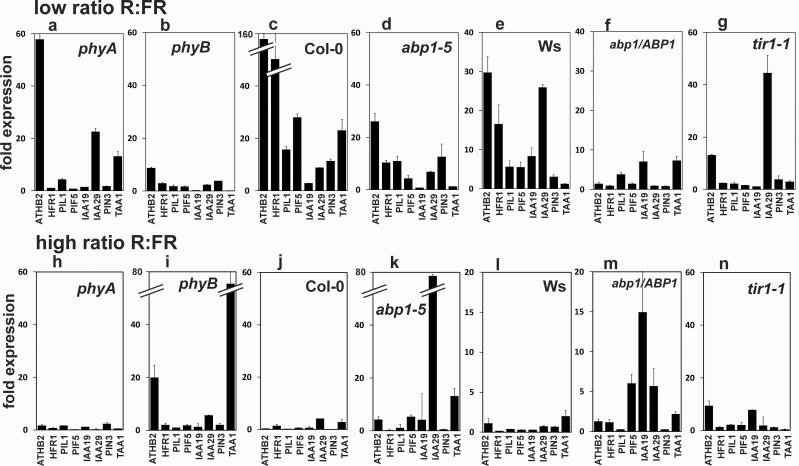
Comparison of regulation of genes by low ratio R:FR (a–f, shade) and high ratio R:FR (g–l, non-shade) in Col, *phyA, phyB, abp1-5*, and *tir1-1*. Seedlings were tested by growing for 3 d in WL and for 1h in WL or white plus added low R:FR ratios or high ratios of R:FR. Expression was normalized to *t*=0 in WL only and set as 1-fold for either genotype. Error bars were calculated according to Pfaffl *et al.* (2002) and are significant when not overlapping (*P* <0.05 or lower). Genotype of *abp1/ABP1* plants was verified by PCR prior to RNA isolation.

To test the hypothesis that ABP1 is involved in the shade-avoidance response, the expression of shade-induced marker genes was quantified after 1h to narrow down the time at which the reorganization of transcription by the interaction of *abp1-5* and phytochromes occurs (Fig. 4a–g). Several FR-light-regulated genes in the shade response (*ATHB2, PIL1, PIF5*, *HFR1*) and of auxin- and light-regulated genes (*IAA19, IAA29, PIN3*) were quantified (Devlin *et al.*, 2003; Salter *et al.*, 2003; Sessa *et al.*, 2005; Roig-Villanova *et al.*, 2006; Tepperman *et al.*, 2006; Hornitschek *et al.*, 2009; Keuskamp *et al.*, 2010; Kunihiro *et al.*, 2011). After 3 d in WL, seedlings were treated for 1h with WL either enriched with FR (low ratio R:FR or shade) where phyB is inactive or with R (high ratio R:FR) where phyB is active (Fig. 4). As a control, seedlings that were treated with WL only were set as 1-fold expression. After only 1h light in shade conditions, expressions of the tested shade marker genes were, in general, higher, consistent with Tepperman *et al.* (2006). In *abp1-5,* induction by shade was about 4–8-fold lower than in Col and in *abp1/ABP1* induction was low compared with Ws. In *phyB*, the induction of expression by 1h low R:FR was 8–15-fold lower than in Col. In *tir1-1*, the induction of *ATHB2* was low and the induction of *IAA29* was higher than in all other genotypes. In *phyA, ATBH2* induction was high and that of *IAA29* was modest and only these two genes were noticeably induced. *ATBH2* and *IAA29* were also induced by low R:FR light in *tir1-1* so that the overall pattern in *tir1-1* was somewhat similar to that in *phyA* but dissimilar to Col.

The expression of the tested genes in high R:FR conditions was generally low or absent in Col or *phyA* (Fig. 4h, j) compared with *abp1-5* and *abp1/ABP1* or the *phyB* mutants (Fig. 4I, k, m) and low in Ws and in *tir1* (Fig. 4l, n). In *abp1-5, abp1/ABP1* or *phyB* several genes at least were induced. Again, this can be interpreted as a decrease in the phyB control of repressing genes in *abp1* mutants similar to that in *phyB* (Jiao *et al.*, 2007). Interestingly, in high R:FR conditions *TAA1* expression, an auxin biosynthesis gene (Tao *et al.*, 2008), was very high in *phyB* (80×) compared with Col, *phyA*, or *tir1* but still high in *abp1-5* (15×) although it was modest in Ws or *abp1/ABP1*. Together, the data suggest that *TAA1* expression is repressed by phyB and repression is absent in shade or in *phyB* seedlings in the high R:FR condition. Regardless of the photoreceptor mechanism, regulation of light-regulated genes was clearly disturbed in *abp1-5, abp1/ABP1*, and *tir1-1*.

## Discussion

Shade avoidance is a complex trait involving inputs from light and hormones, especially auxin. The shade-avoidance response is induced in plants by sensing a low R:FR ratio in the WL background. The shade-avoidance response is primarily sensed by phyB (Reed *et al.*, 1993) induced by a low R:FR ratio, although phyD and phyE participate to some degree in sensing (Aukerman *et al.*, 1997; Devlin *et al.*, 1998; Devlin *et al.*, 1999). Low signalling activity by CRY1 in low B light also contributes to the shade-avoidance response (Ballaré, 2009; Kunihiro *et al.*, 2010). Our physiological results and our results on auxin-induced gene expression (Fig. 1; see Supplementary Fig. S3 at *JXB* online) show that *abp1-5* is an auxin signalling mutant just as is *abp1/ABP1* (Effendi *et al.*, 2011) and both have the capacity to modulate red light responses.

**Fig. 5.**
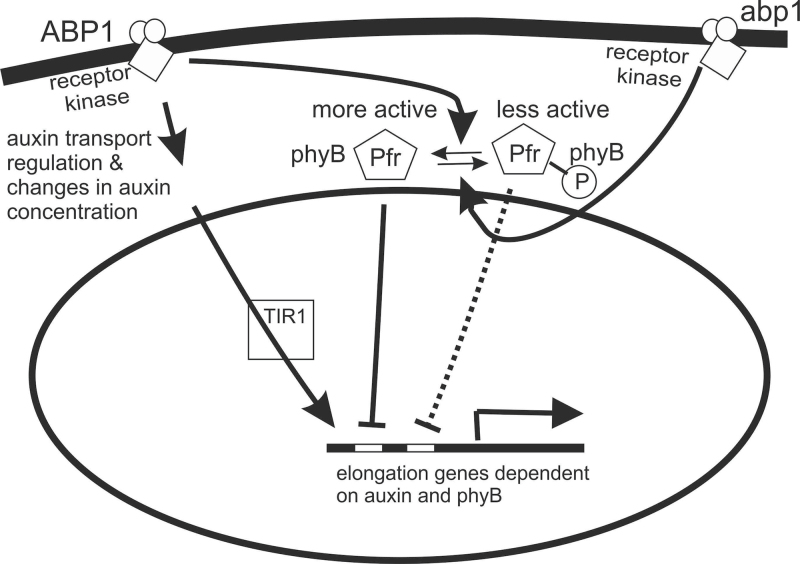
A working model of the functional interaction of ABP1 and phytochrome B. ABP1 interacts with a postulated transmembrane docking protein (Klämbt, 1990; Scherer *et al.*, 2012) capable of transmitting the auxin signal across the membrane. This could be a receptor kinase or a calcium channel (or other) so that a post-translational modification of phyB as described by Medzihradsky *et al.* (2013) seems a possibility for functional interaction. According to the authors, comparison of *phyB-9* plants expressing phospho-mimic yellow fluorescent fusion protein phyB^Ser86Asp^-YFP or nonphosphorylatable phyB^Ser86Ala^-YFP demonstrated that phosphorylation of Ser-86 negatively regulates all physiological phyB responses tested by them including the response to shade. Light-independent relaxation of the phosphomimicking phyB^Ser86Asp^ Pfr into phyB^Ser86Asp^ Pr (dark reversion) is strongly enhanced both *in vivo* and *in vitro*. Faster dark reversion attenuates red light-induced nuclear import and interaction with the negative regulator PHYTOCHROME INTERACTING FACTOR3 compared with the wild-type version phyB^Ser86^-GFP (Medzihradsky *et al.*, 2013). It is suggested that ABP1 can influence this phosphorylation–dephosphorylation equilibrium towards the more active form. This more active form can still be inactivated by FR so that wt ABP1 plants show a small elongation to shade whereas the *abp1* mutants show a hyper-response.

Based on published observations (Shinkle and Jones, 1988; Jones *et al.*, 1991; Shinkle *et al.*, 1992, 1998; Barker-Bridges *et al.*, 1998; Robert *et al.*, 2010; Xu *et al.*, 2010; Effendi *et al.*, 2011) and the data presented here, it is illustrated in Fig. 5 that one important nexus linking auxin and R signalling is ABP1. Since ABP1 is not a cytoplasmic protein, any direct interaction with phyB is unexpected. However, ABP1-mediated auxin signalling through the aforementioned ABP1 docking protein and downstream factors may regulate phyB-dependent signalling. Inhibition of the growth repressing regulatory activity of phyB is the predominant mechanism.

### ABP1 and predominantly phyB link auxin and red light physiology

Increased elongation in low ratio R:FR light is a hallmark of the response of plants to physiological shade and low signalling output in this light by phyB is recognized to be the main reason (Reed *et al.*, 1993; Stamm and Kumar, 2010). The *tir1* alleles did not respond to low ratio R:FR conditions (Fig. 2). With respect to hypocotyl elongation *abp1-5* and *abp1/ABP1* resemble weak *phyB* mutants (Fig. 2) in that they hyperelongate in low ratio R:FR conditions compared with the shade responses of their wild types. However, the insensitivity to R as seen in the response of *phyB* to high ratio R:FR was not observed in them.

NPA applied under red light revealed that *abp1* mutants phenocopy phytochrome mutants in their loss of gravitropic orientation (Fig. 3). Hypocotyl gravitropism requires asymmetrical auxin transport (Friml *et al.*, 2002; Nagashima *et al.*, 2008*a*, *b*). Gravitropism is inhibited by R and FR and thus phyB and phyA are the relevant photoreceptors identified in continuous R or FR light (Liscum and Hangarter, 1993; Robson and Smith, 1996). Inhibition of hypocotyl gravitropism by phytochromes in our experiments was evidenced by a comparison of *phyA* and *phyB* seedlings with the *abp1* mutants with and without NPA (Fig. 3). We did not use R or FR alone but with added WL all genotypes grew without NPA almost completely upright and any red light effect was small. Increased randomization of hypocotyls in *phyA, phyB*, and *abp1* mutant lines in the presence of NPA indicated that *abp1* mutants, in general, behaved as weak phenocopies of phytochrome-deficient seedlings (Fig. 3). Whether phyA or phyB or signalling from both phytochromes was affected in the *abp1* mutants cannot be decided but, clearly, auxin transport was disturbed in this loss of gravitropic orientation and NPA acted as an enhancer. Although PIN proteins are known to regulate gravitropism and expression analysis of the DR5:GUS auxin reporter gene in *pin3* seedlings suggested that they are impaired in the normal lateral transport during tropism (Friml *et al.*, 2002), it is clear that NPA also impairs the asymmetric distribution of auxin in hypocotyl tropism in an ABCB19-dependent manner (Nagashima *et al.*, 2008*b*). The proteins actually binding NPA are the ABCB transporters (Bailly *et al.*, 2011). ABCB19 transporter mutants are agravitropic (Noh *et al.* 2001; Blakeslee *et al.* 2007; Nagashima *et al.* 2008*b*) and in red light their hypocotyl orientation randomizes (Nagashima *et al.*, 2008*a*). PIN proteins act co-operatively with ABCB proteins (Blakeslee *et al.*, 2007; Bailly *et al.*, 2011) so that PINs in tropisms may also act in a co-operative manner with the ABCB auxin transporters. In monochromatic R light ABCB19 and ABCB1 protein expression decreases (Nagashima *et al.*, 2008*a*, *b*). Adding NPA in our experiments probably further reduced their activity leading to strong randomization. In conclusion, auxin transport components and red light sensors interact in the inhibition of hypocotyl gravitropism and this interaction is disturbed in *abp1* mutants pointing out an ABP1 and phytochrome interaction.

### *Light-induced gene expression in* abp1 *mutants*

Expression patterns of known shade-induced genes in low ratio R:FR (shade) and high ratio R:FR light support our hypothesis that ABP1 and phyB are linked in red light signalling. Since kinetics is so important in the argument, it is noteworthy that this difference could be detected as early as one hour after the start of shade. At about this time point, shade-induced elongation starts to become apparent (Cole *et al.*, 2011; Li *et al.*, 2012).

Our hypothesis of functional interaction of ABP1 and phyB is further supported by data on the expression of shade-induced genes. Compared with Col, the induction of the shade marker genes (*ATHB2, HFR1, PIF1, PIF5)* is much lower in *abp1-5* and very low in *phyB* and also low in *abp1/ABP1* compared with Ws (Fig. 4). *IAA19* and *PIN3,* both of which are induced by auxin and (to a low extent) in shade in wt and *abp1-5*, were not induced in *abp1/ABP1.* Lack of expression of shade-induced genes in high ratio R:FR demonstrate that, in Col and *phyA* (being wt with respect to phyB), expression was mostly repressed but in *phyB*, *abp1-5*, and *abp1/ABP1* some genes escaped light repression (Fig. 4h–n).

The *tir1-1* mutant showed altogether a different pattern of regulation of light-induced genes than either Col, *abp1-5,* or *abp1/ABP1* with none of the genes tested here being induced by shade except *IAA29* (Fig. 4). While *IAA29* was highly induced in *tir1* compared with Col or *abp1* mutants, IAA29 is not ubiquitinated by TIR1 (Dreher *et al.*, 2006; Maraschin *et al.*, 2009) so it might escape control by TIR1 in *tir1*. Defects in the co-regulation of genes induced by light and by auxin, as noted before (Devlin *et al.*, 2003; Kunihiro *et al.*, 2011; Stamm and Kumar, 2010), is a possibility that could explain this lack of shade response in the *tir1* mutant and the hyperelongation of *abp1* mutants. The lack of shade-induced elongation in *tir1* probably indicates the necessity of activation of further auxin-regulated genes than those tested here for sustained elongation and other members of the TIR/AFB family may act redundantly with TIR1 in this.

### Auxin biosynthesis, auxin transport, and shade response

One important auxin input into the shade-avoidance response is an increase in auxin signal strength by the shade-dependent induction of *TAA1* transcription, an auxin biosynthesis gene (Tao *et al.*, 2008). Our findings confirm this for low R:FR light in the wild type but, in *abp1-5*, *TAA1* is only induced a little in *abp1/ABP1* (Fig. 4) and not at all in *phyB* and *tir1*. This does not correlate with the hypocotyl lengths of these genotypes in shade light. Further, how the early timing of the transcriptional response of *TAA1* translates into an increase of IAA is still unclear (Quint *et al.*, 2009; Mashiguchi *et al.*, 2011; Mana and Nemoto, 2012). So it is also unclear how exactly auxin concentration makes its input into the shade responses (Stamm and Kumar, 2010; Nozue *et al.*, 2011).

A *potential* shared element of auxin and phyB signalling in the shade-avoidance syndrome may be PIN3 (Keuskamp *et al.*, 2010; Kozuka *et al.*, 2010). As discussed above PIN3 and ABCB transporters probably co-operatively participate in their responses to auxin and light (Blakeslee *et al.*, 2007; Nagashima *et al.*, 2008*a*; Bailly *et al.*, 2011). Rapid regulation of ABCB is not so well investigated as that of PIN proteins. The regulation of PIN proteins may occur as protein subcellular re-distribution most rapidly (Kleine-Vehn and Friml, 2008) and/or at the transcriptional level (Vieten *et al.*, 2005; Effendi and Scherer, 2011). Auxin modulates auxin transport within a few minutes independently of transcriptional regulation (Paciorek *et al.*, 2005; Petrasek *et al.*, 2006; Robert *et al.*, 2010). Specifically, the regulation of *PIN3* and perhaps other *PIN* genes could be part of a common set of intermediates between ABP1 and phyB. Consistent with this notion, the expression of *ABCB19* is repressed by R although modes of interaction in shade of ABCB19 and PIN3 are unknown (Nagashima *et al.*, 2008*a*, *b*). ABP1 regulates polar auxin transport at the organ level (Effendi *et al.*, 2011) and by the regulation of *PIN3* expression (Effendi and Scherer, 2011). Exactly how phyB (or/and phyA) enters into the ABP1 pathway remains mostly unclear. However, recently Medzihradzky *et al.* (2013) showed that phosphorylation of phyB inhibits light-induced signalling. The transmembrane protein postulated by Klämbt (1990) to interact with ABP1 could have the necessary enzymatic activity, for example, a protein kinase or a calcium channel stimulating calcium-dependent phosphorylation, to transmit a phosphorylation as the signal to activate phyB. Our working model (Fig. 5) presented here provides a launching point to dissect the recently-speculated cytosolic phytochrome signalling pathway (Rösler *et al.*, 2010).

## Supplementary data

Supplementary data can be found at *JXB* online.

Supplementary Fig. S1. Auxin sensitivity of *abp1-5*.

Supplementary Fig. S2. Flowering date in Col-0 and *abp1-5* plants grown in short days (16/8h L/D).

Supplementary Fig. S3. Rapid regulation of early auxin genes by 10 μM 1-NAA in Col-0 wild type and *abp1-5* mutant seedlings.

Supplementary Fig. S4. Spectra used in the shade-avoidance experiments.

Supplementary Fig. S5. Effect of NPA on elongation in (A) low ratio (R:FR) supplemented WL or (B) in high ratio (R:FR) supplemented WL light.

Supplementary Table S1. List of primers.

Supplementary Data

## Abbreviations:

ABP1: AUXIN-BINDING PROTEIN1
B: blue
FR: far red
NPA: naphthylphthalamic acid
PIN: PIN-FORMED protein
R: red
TIR1: TRANSPORT-INHIBITOR-RESPONSE1
WL: white light
wt: wild type.
