# Supplementary material for: AUXIN-BINDING-PROTEIN1 (ABP1) in phytochrome-B-controlled responses
Source: J Exp Bot. 2013 Sep 19;64(16):5065–74. doi: 10.1093/jxb/ert294 (PMC3830486; doi:10.1093/jxb/ert294)
Supplement: Supplementary Data [file supp_64_16_5065__index.html]

AUXIN-BINDING-PROTEIN1 (ABP1) in phytochrome- B-controlled responses — AUXIN-BINDING-PROTEIN1 (ABP1) in phytochrome-B-controlled responses — Supplementary Data 

# AUXIN-BINDING-PROTEIN1 (ABP1) in phytochrome-B-controlled responses

## Supplementary Data

Data files

**Files in this Data Supplement:**

- Supplementary Data - Supplementary Data
