# Supplementary material for: AUXIN-BINDING-PROTEIN1 (ABP1) in phytochrome-B-controlled responses
Source: J Exp Bot. 2013 Sep 19;64(16):5065–74. doi: 10.1093/jxb/ert294 (PMC3830486; doi:10.1093/jxb/ert294)
Supplement: Supplementary Data [file supp_ert294_jexbot102574_file001.pdf]

Supporting information Effendi Y, Jones AM, Scherer GFE. 2011

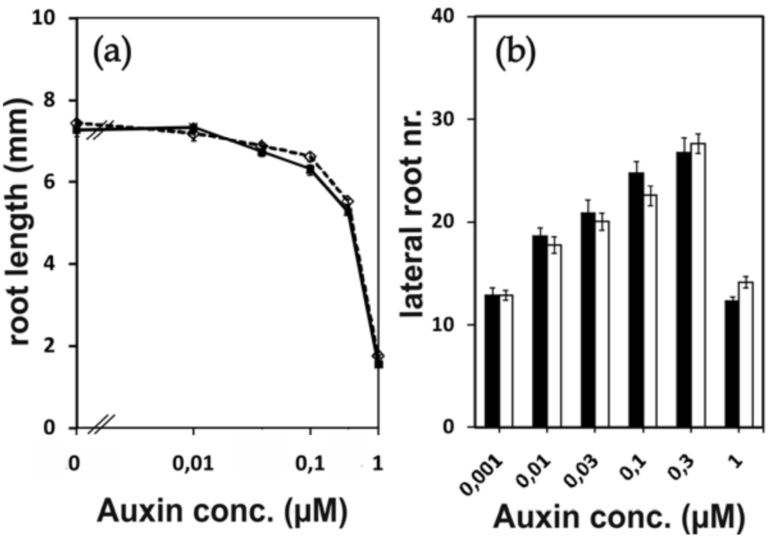

Fig. 1S. Auxin sensitivity of *abp1-5*. (a) Root length of 10 days old light-grown Col-0 (black squares) and *abp1-5* (diamonds) seedlings. (S.E., n=30). (b) Lateral root formation in response to 1-NAA in 10 days old light-grown Col-0 (black bars) and *abp1-5* (white bars) seedlings. (S.E., n=30).

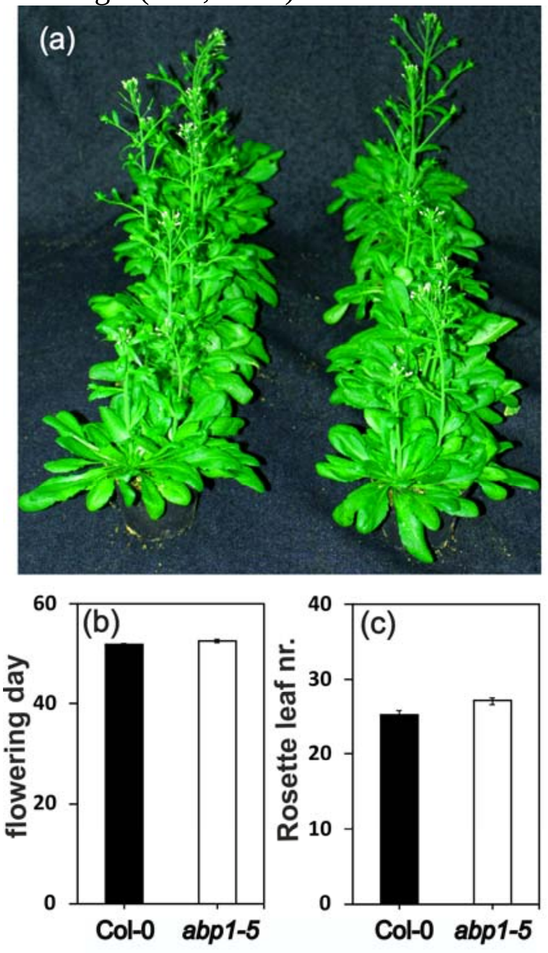

Fig. 2S. Flowering date in Col-0 and *abp1-5* plants grown in short days (16/8 L/D). (a) Representative groups of plants at day 52. Left: Col-0; right: *abp1-5*. (b) Day of appearance of first flower. (c) Number of rosette leaves at the day of flowering. (S.E.; n=33).

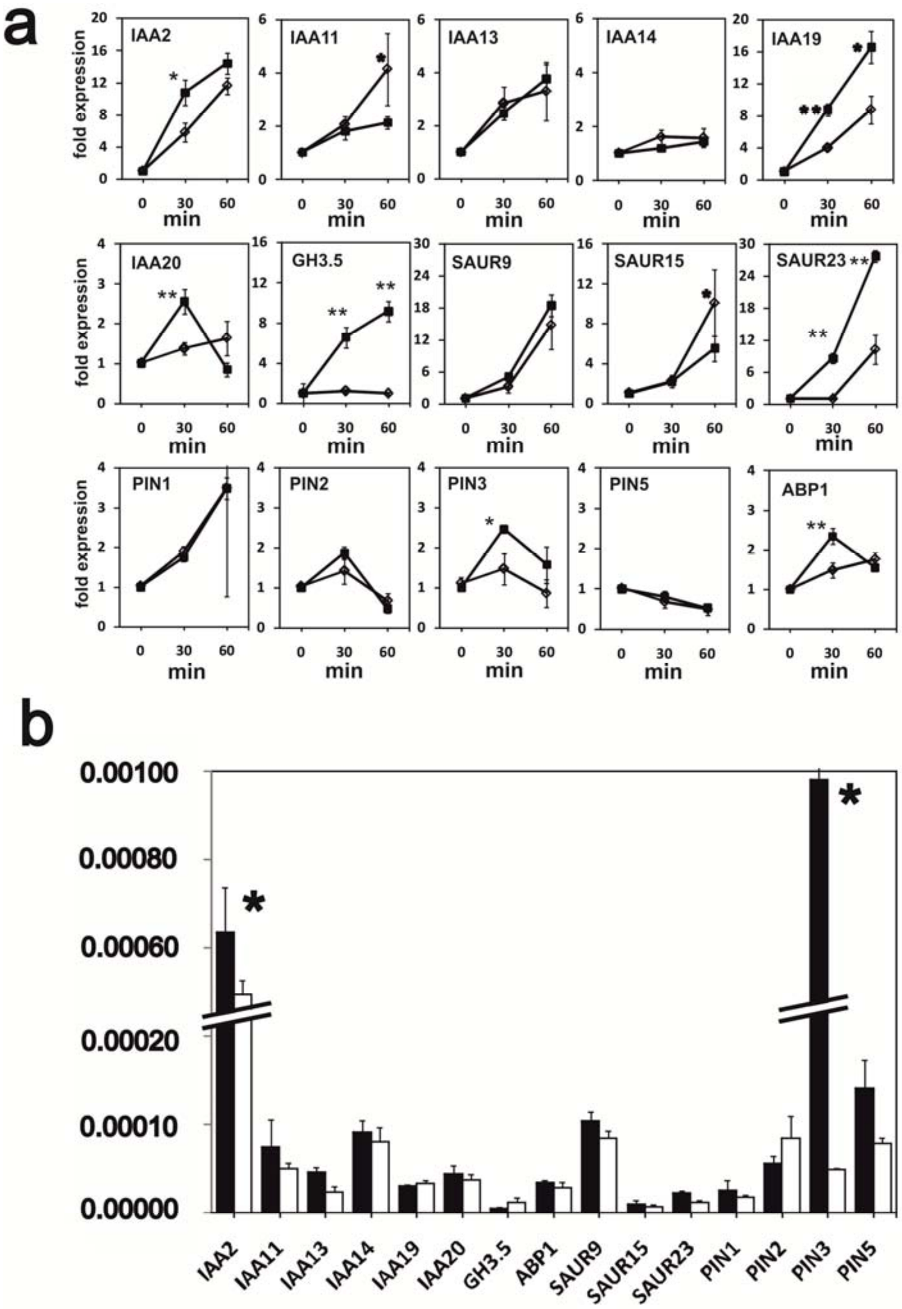

Fig. 3S. Rapid regulation of early auxin genes by 10 µM 1-NAA in Col-0 wild type and *abp1-5* mutant seedlings. (a) Seedlings were grown for 14 days in WL of about 50 µmoles$\cdot$m^{-2}sec^{-1}. Expression was normalized to t=0 set as 1-fold for either genotype. Black squares: wild type Col-0; diamonds: *abp1-5*. Significance levels in (A): *: p<0.05; **: p<0.01; ***: p<0.001. (b) Comparison of "basal" expression levels in *abp1-5* to Col at t=0. Expression of the reference gene 18S in wild type Col was set as 1-fold. Black bars: wild type Col-0; white bars: *abp1-5*.

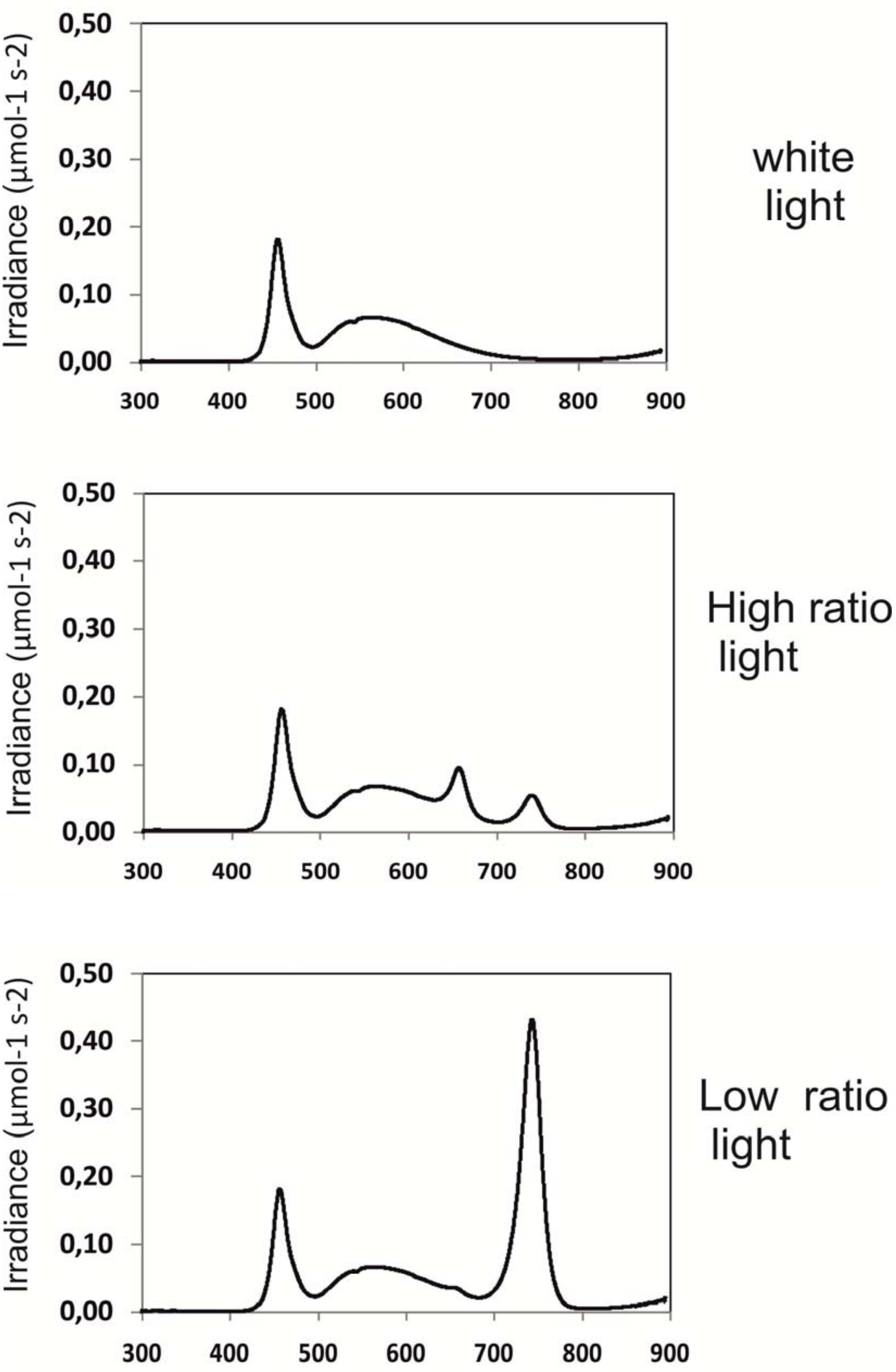

Fig. 4S. Spectra used in the shade avoidance experiments. (A) White LEDs only. (B) White LEDs with added red and far red LEDs to generate a high ratio of R:FR of 1,106. (C) White LEDs with added red and far red LEDs to generate a low ratio R:FR of 0,098. (ratio: 665-675nm/725-735nm). Spectra were measured with a spectrometer Ocean Optics "USB4000" equipped with Cosinus corrector and the software Spectra Suite.

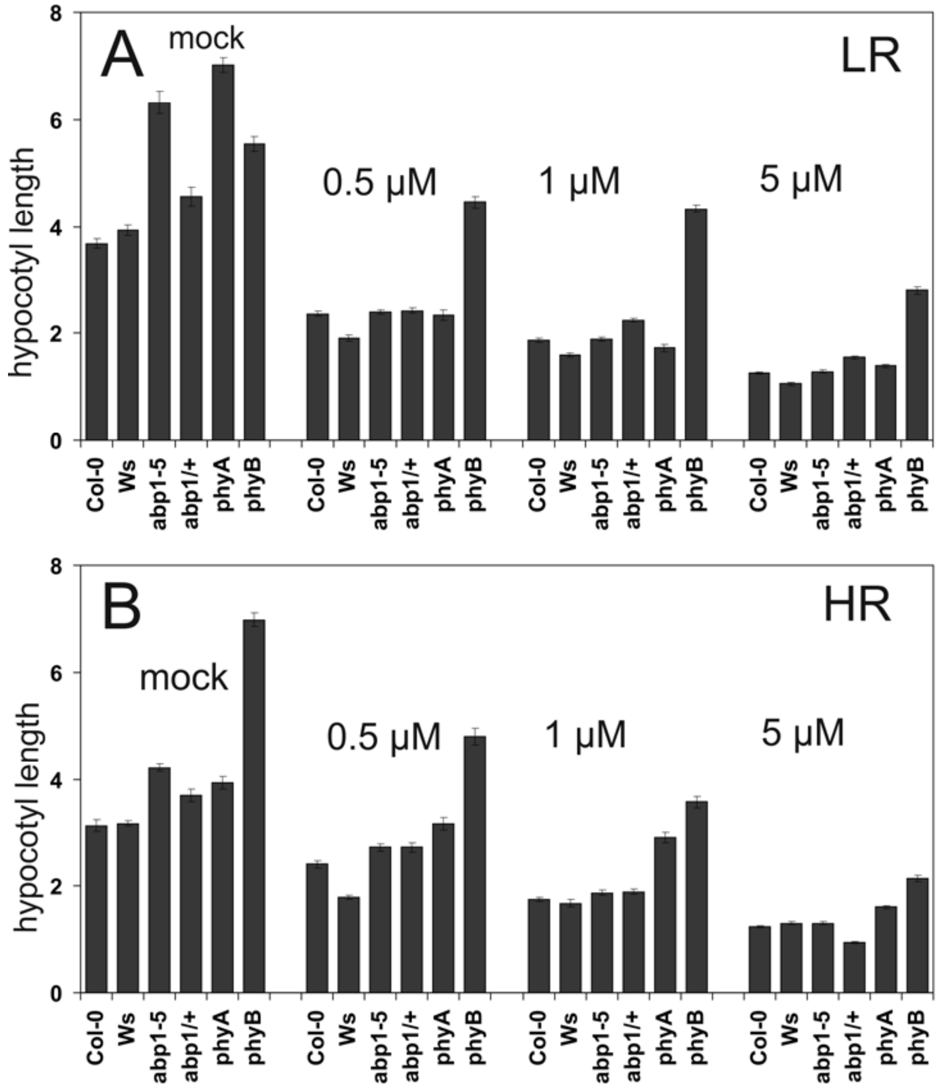

Fig. 5S. Effect of NPA on elongation in (A) low ratio (R:FR) supplemented WL or (B) in high ratio (R:FR) supplemented WL light. Seedlings were pre-grown in WL for 3 d on ½ MS plates containing NPA as indicated or mock, then kept in the indicated light conditions for another 3 d. Data are from 24-56 seedlings per assay (S.E.). Genotype of *abp1/ABP1* plants was verified by PCR and only *abp1/ABP1* plants were counted.

Supplemental Table 1. List of primers.

Light experiments:
*ATHB2*_F 5’-GAG GTA GAC TGC GAG TTC TTA CG 3’
*ATHB2*_F 5’-GCA TGT AGA ACT GAG GAG AGA GC-3’
*HFR*_F 5’-CAC AAG ACG GAC AAG GTT TCG-3’
*HFR*_R 5’-GTC AGC ATG TGG TTG TGC ATT C-3’
*PIL1*_F 5’-TGG TGC CTT CGT GTG TTT CTC A-3’
*PIL1*_R 5’-GGA CGC AGA CTT TGG GAA TTG-3’
*PIF5*_F 5’-GAT GCA GAC CGT GCA ACA AC-3’
*PIF5*_R 5’-CTT TTA TGC TTG CTT AGG CG-3’
TAA1-forw 5‘-TGGATCATGGTGATCCAACG-3’;
TAA1-rev 5’-GCTCAAGGAACCAACACAAG-3’

Auxin treatment:

18S rRNA forw 5'-GGC TCG AAG ACG ATC AGA TAC C-3';
18S rRNA rev 5'-TCG GCA TCG TTT ATG GTT-3';
ABP1 forw 5'-ACG AGA AAA TCA TAC CAA TTC GGA CTA ACC-3';
ABP1 rev 5'-GTA TCT ACG TAG TGT CAC AAA ACC TCA AC-3';
IAA2 forw 5'-GGT TGG CCA CCA GTG AGA TC-3';
IAA2 rev 5'-AGC TCC GTC CAT ACT CAC TTT CA-3';
IAA11 forw 5'-CCT CCC TTC CCT CAC AAT CA-3';
IAA11 rev 5'-AAC CGC CTT CCA TTT TCG A-3';
IAA12 forw 5’ CGT TGG GTC TAA ACG CTC TG 3’;
IAA12 rev 5’ TTC CGC TCT TGC TGC CTT CA 3’;
IAA13 forw 5'-CAC GAA ATC AAG AAC CAA ACG A-3';
IAA13 rev 5'-CAC CGT AA CGT CGA AAA GAG ATC-3';
IAA14 forw 5'-CCT TCT AAG CCT CCT GCT AAA GCA C-3';
IAA14 rev 5'-CCA TCC ATG GAA ACC TTC AC-3';
IAA19 forw 5'-GGT GAC AAC TGC GAA TAC GTT ACC-3';
IAA19 rev 5'-CCC GGT AGC ATC CGA TCT TTT CA-3';
IAA20 forw 5'-CAATATTTCAACGGTGGCTATGG-3';
IAA20 rev 5'-GCC ACA TAT TCC GCA TCC TCT A-3';
GH3.5 forw 5'-AGC CCT AAC GAG ACC ATC CT-3;
GH3.5 rev 5'-AAG CCA TGG ATG GTA TGA GC-3';
SAUR9 forw 5'-GAC GTG CCA AAA GGT CAC TT-3';
SAUR9 rev 5'-AGT GAG ACC CAT CTC GTG CT-3';
SAUR15 forw 5'-ATG GCT TTT TTG AGG AGT TTC TTG GG-3;
SAUR15 rev 5'-TCA TTG TAT CTG AGA TGT GAC TGT G-3';
SAUR23 forw 5'-ATG GCT TTG GTG AGA AGT CTA TTG GT-3;
SAUR23 rev 5'-TCA ATG GAG CCG AGA AGT CAC ATT GA-3'.
PIN1-forw: 5’GGA GAC TTA AGT AGG AGC TCA GCA-3’;
PIN1-rev: 5’-CCA AAA GAG GAA ACA CGA ATG-3’;
PIN2-forw: 5’-TAT CAA CAC TGC CTA ACA CG-3’;
PIN2-rev: 5’-GAA GAG ATC ATT GAT GAG GC-3’;
PIN3-forw: 5’-GAG TTA CCC GAA CCT AAT CA-3’;
PIN3-rev: 5’-TTA CTG CGT GTC GCT ATA GT-3’;
PIN5-forw: 5’-ACC CTG CCG CTC TTC ACC A-3’;
PIN5-rev: 5’-GCC CAC AAC GCT AAG ACC G-3’;
